# Ligation-assisted mucosal unroofing and snare resection: a simplified and cost-effective technique for gastric submucosal tumors

**DOI:** 10.1055/a-2901-6737

**Published:** 2026-07-13

**Authors:** Qizheng Lu, Peng Xie, Qinyi Liu, Shaoyan Guo, Lifang Ye, Jiachuan Wu

**Affiliations:** 1 Department of Digestive MedicineThe Affiliated Guangdong Second Provincial General Hospital of Jinan UniversityGuangzhouGuangdongChina

## A case description



**Video 1** Ligation-assisted mucosal unroofing and snare resection of a gastric submucosal tumor arising from the muscularis propria using standard polypectomy equipment.


Endoscopic submucosal excavation (ESE) is a well-established treatment for gastric submucosal tumors (SMTs). 1​ ​2​ However, for small lesions (8–15 mm), ESE remains technically demanding, time-consuming, and resource-intensive, often associated with a steep learning curve. 3​ ​4​ Therefore, a standardized and more accessible strategy is clinically warranted. Herein, we demonstrate a novel technique that effectively addresses these limitations, requiring only standard polypectomy equipment ( Video 1 ).

**Fig. 1:**
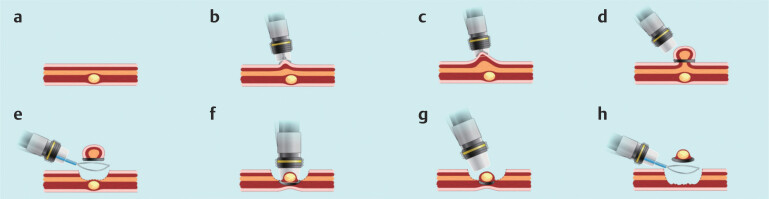
Schematic representation of the novel SMT endoscopic technique. ( **a** ) Tumor. ( **b** ) Grasping the overlying mucosa. ( **c** ) Retracting the mucosa into the cap. ( **d** ) Ligation of the overlying mucosa. ( **e** ) Incision of the ligated mucosa. ( **f** ) Exposure of the underlying tumor. ( **g** ) Ligation of the tumor. ( **h** ) En bloc resection of the tumor.

**Fig. 2:**
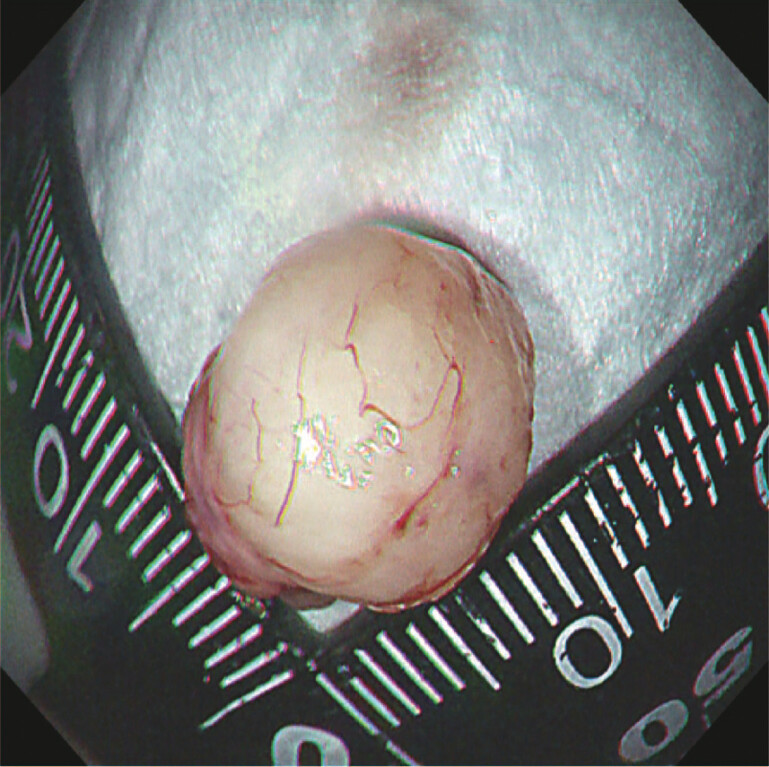
En bloc resection of the gastric submucosal tumor measuring 12 mm×10 mm.

The initial steps involved marking the tumor ( Fig. 1a ) margins and mounting the over-the-scope band ligator (SureFire, MBLS-6, MICRO-TECH, Nanjing, China). The mucosa overlying the SMT was grasped with forceps ( Fig. 1b ) and retracted into the cap ( Fig. 1c ), allowing for successful ligation ( Fig. 1d ). A forceps-assisted approach ensures targeted capture without involving adjacent tissues or damaging the tumor body. A snare was then utilized to incise the ligated mucosa using electrocautery ( Fig. 1e ), effectively creating a mucosal window to expose the underlying tumor ( Fig. 1f ). Unroofing allows the tumor to be aspirated under direct vision, facilitating easier and more accurate ligation of the tumor base. Subsequently, the ligator was reapplied to capture the denuded tumor, deploying a band precisely at its base to ensure mechanical isolation ( Fig. 1g ). Finally, en bloc resection of the tumor was then achieved via snare electrocautery ( Fig. 1h ). In our case, the measured specimen size is about 12 mm×10 mm ( Fig. 2 ).

In our practice, this approach required an average procedural time of only 10 to 15 minutes, yielding an uneventful postoperative course without complications. By obviating the need for specialized electrosurgical knives and advanced dissection skills, this technique significantly minimizes procedural costs and flattens the learning curve. Leveraging fundamental banding and polypectomy proficiency, it offers a safe, efficient, and readily adoptable strategy for gastric SMT treatment, particularly in non-tertiary centers.

Endoscopy_UCTN_Code_TTT_1AO_2AG
